# Preventing acute liver injury via hepatocyte‐targeting nano‐antioxidants

**DOI:** 10.1111/cpr.13494

**Published:** 2023-05-04

**Authors:** Xuejiao Yuan, Yanfeng Zhou, Jinli Sun, Shanshan Wang, Xingjie Hu, Jiyu Li, Jing Huang, Nan Chen

**Affiliations:** ^1^ College of Chemistry and Materials Science, The Education Ministry Key Lab of Resource Chemistry, Joint International Research Laboratory of Resource Chemistry of Ministry of Education, Shanghai Key Laboratory of Rare Earth Functional Materials, and Shanghai Frontiers Science Center of Biomimetic Catalysis Shanghai Normal University Shanghai China; ^2^ School of Public Health Shanghai Jiao Tong University School of Medicine Shanghai China; ^3^ He'nan Xibaikang Health Industry Co., Ltd Jiyuan China; ^4^ Department of Neurology Xuhui District Central Hospital Shanghai China

## Abstract

Acute liver injury (ALI) is a severe liver disease that is characterized by sudden and massive hepatocyte necrosis and deterioration of liver functions. Oxidative stress is increasingly recognized as a key factor in the induction and progression of ALI. Scavenging excessive reactive oxygen species (ROS) with antioxidants has become a promising therapeutic option, but intrinsically hepatocyte‐targeting antioxidants with excellent bioavailability and biocompatibility are yet to be developed. Herein, self‐assembling nanoparticles (NPs) composed of amphiphilic polymers are introduced to encapsulate organic Selenium compound L‐Se‐methylselenocysteine (SeMC) and form SeMC NPs, which protect the viabilities and functions of cultured hepatocytes in drug‐ or chemical‐induced acute hepatotoxicity models via efficient ROS removal. After further functionalization with the hepatocyte‐targeting ligand glycyrrhetinic acid (GA), the resultant GA‐SeMC NPs exhibit enhanced hepatocyte uptake and liver accumulation. In mouse models of ALI induced by acetaminophen (APAP) or carbon tetrachloride (CCl_4_), treatment with GA‐SeMC NPs significantly decrease the levels of hepatic lipid peroxidation, tissue vacuolization and serum liver transaminases, while prominently increase that of endogenous antioxidant enzymes. Our study therefore presents a liver‐targeting drug delivery strategy for the prevention and treatment of hepatic diseases.

## Graphical abstract

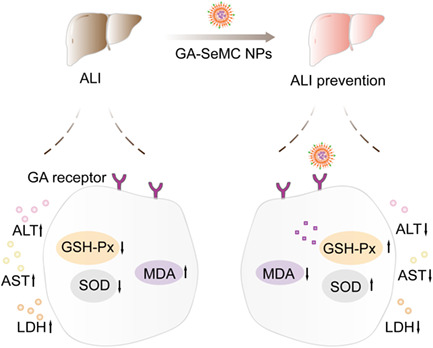

## 1 INTRODUCTION

Acute liver injury (ALI), defined as sudden and massive hepatocyte necrosis and subsequent deterioration of liver functions, is a type of lethal disease worldwide.^1^, ^2^, ^3^ Clinically, drug overdose and accidental toxic reagent ingestion are the main causes of ALI.^4^, ^5^, ^6^, ^7^ Currently, available preventive and therapeutic strategies for these chemical‐induced liver injuries are still limited.^8^, ^9^ It is noteworthy that overproduction of reactive oxygen species (ROS) is found in the liver of ALI mouse models and patients, and oxidative stress is demonstrated to be a key mechanism under hepatotoxicity.^6^, ^10^, ^11^, ^12^ Therefore, scavenging excessive ROS has been considered as an accessible solution to improve the microenvironment of the injured liver and alleviate the pathological progression. Broad‐spectrum antioxidants such as N‐acetyl cysteine have exhibited preventive and therapeutic effects on ALI.^10^, ^13^ However, the fast excretion, unsatisfactory accumulation at the injury foci, and the resulting poor bioavailability largely limit their clinical applications.^10^, ^14^ It is therefore highly desirable to develop more effective strategies focusing on liver‐targeted ROS scavenging.

Selenium (Se), an essential trace mineral, is a critical structural and catalytic component of the antioxidant enzymes (e.g., glutathione peroxidase [GSH‐Px]) that protect the cell from oxidative damage, playing important roles in maintaining human health.^15^, ^16^, ^17^ Supplementation of Se has been shown to alleviate the tissue and organ damage induced by various toxic substances, including heavy metal ions, mycotoxins, antitumor drugs, etc.^18^, ^19^, ^20^, ^21^, ^22^ Among various forms of Se compounds, the inorganic Se compounds are abundant and inexpensive, but exhibit low absorption and conversion rates as well as higher toxicity than organic Se compounds.^18^, ^23^, ^24^ L‐Se‐methylselenocysteine (SeMC), a new generation of organic Se compound, has shown protective effects on the organ damage caused by chemotherapy‐induced systemic toxicity.^25^ However, the hepatoprotective effects of SeMCs against liver injury are not well explored.

In recent years, the advent and rapid development of nanotechnology has offered great opportunities for drug administration.^26^, ^27^, ^28^, ^29^, ^30^, ^31^, ^32^ The resulting concept of ‘nanomedicine’ has enabled the development of new ROS scavenging strategies against ROS‐related diseases using various functional nanomaterials.^14^, ^33^, ^34^ Nanoencapsulation of antioxidants by engineering amphiphilic micellar nanoparticles (NPs) has attracted great interest for the researchers due to their targeting properties and sustained‐releasing features, which was regarded as a breakthrough in increasing the bioavailability and therapeutic efficacy of the antioxidants while reducing their potential side effects.^35^, ^36^ Inspired by the advantages of amphiphilic micellar NP‐based nanomedicine, nanoformulated SeMCs that were encapsulated by amphiphilic molecules and glycyrrhetinic acids (GA), namely GA‐SeMC NPs, were generated in this study. We found that GA‐SeMC NPs showed higher hepatocyte uptake and liver accumulation. In mouse models of ALI, these nano‐antioxidants efficiently suppressed APAP‐ or CCl_4_‐induced increases in the levels hepatic oxidative stress, histological lesions and serum transaminases, and promoted the expression of antioxidant enzymes, exhibiting superior hepatoprotective effects.

## 2 MATERIALS AND METHODS

### 2.1 Materials and cell culture

Normal liver cell line L‐02 was purchased from the Cell Bank of Chinese Academy of Sciences (Shanghai) and was maintained in RPMI 1640 medium (Gibco) supplemented with 10% foetal bovine serum (FBS, from Gibco) and antibiotics (streptomycin and penicillin, 100‐U/ml each, Invitrogen). DSPE‐PEG_2000_‐OH, DSPE‐PEG_2000_‐NH_2_, PCL_1000_‐PEG_2000_‐NH_2_, PCL_2000_‐PEG_5000_‐NH_2_, PCL_5000_‐PEI_2000_ copolymers were purchased from Xi'an ruixi Biological Technology Co., Ltd. 18β‐Glycyrrhetinic acid was purchased from MedChemExpress (Shanghai). SeMC was provided by He'nan Xibaikang Health Industry Co., Ltd. Cell Counting Kit‐8 (CCK‐8) was purchased from Beyotime. 2′,7′‐dichlorofluorescin diacetate (DCFH‐DA), Rhodamine B, acetaminophen (APAP) and carbon tetrachloride (CCl_4_) were purchased from Sigma–Aldrich.

### 2.2 Synthesis and characterization of SeMC NPs

SeMC (5 mg, 27 μmol) and DSPE‐PEG‐NH_2_ (10 mg, 3.6 μmol) were dissolved with Milli‐Q water and CH_2_Cl_2_, respectively. The two solutions were mixed and then transferred into a round‐bottom flask. The solvent was subsequently evaporated by rotary evaporation to obtain a thin film. After that, the film was hydrated at 37°C for 2 h with 1 mL HEPES‐buffered saline (pH 7.4, 10 mmol/L), and then a clear SeMC NP solution was obtained. The unloaded polymeric nanoparticles were prepared by the above‐described procedures without SeMC.

The transmission electron microscopy (TEM, JEOL 2100) was utilized to characterize the surface morphology of the SeMC NPs. The hydrodynamic diameter and zeta potential measurements were carried out using a ZEN3690 Zetasizer (Malvern). The in vitro release profiles of SeMCs from SeMC NPs were studied by dialytic diffusion method. In brief, 2 mL SeMC NPs were added to a dialysis bag (molecular weight, 3500 Da) in phosphate buffered solutions (PBS), pH 5.5 and 7.4, and the dialysis bag was placed in a large beaker containing 30 mL PBS at 37°C with magnetically controlled agitation. The release medium outside the dialysis bag was replaced with fresh PBS, and the release medium was detected by ninhydrin method. The concentration of released drug was determined by measuring the absorbance intensity at 570 nm.

### 2.3 Synthesis and characterization of GA‐SeMC NPs

GA‐SeMC NPs were synthesized via a two‐step procedure. First, GA (0.4707 g, 1 mmol), EDC (0.7668 g, 4 mmol), and NHS (0.4604 g, 4 mmol) were mixed in 4 mL CH_2_Cl_2_, then the solution was stirred and incubated at room temperature for 5 h to activate the terminal carboxyl group of GA. Then, the reaction solution was dropped slowly into DSPE‐PEG‐NH_2_ (10 mg, 3.6 μmol) solution in 2 mL CH_2_Cl_2_, stirred and incubated at room temperature for 24 h, DSPE‐PEG‐GA was obtained. Second, DSPE‐PEG‐NH_2_ (10 mg, 3.6 μmol), SeMCs (5 mg, 27 μmol) were dissolved in 2 mL CH_2_Cl_2_. DSPE‐PEG‐GA, DSPE‐PEG‐NH_2_, and SeMCs were then added together to a round‐bottom flask, the solvent was then evaporated to remove CH_2_Cl_2_, and a lipid film was formed using a rotary evaporator. The lipid film was subsequently hydrated with 4 mL phosphate buffer (PBS) buffer (pH 7.4) for 2 hours at ambient temperature to form GA‐SeMC NPs. The particle sizes and zeta potentials of GA‐SeMC NPs were measured by nano‐analyser. The structure of the complex was analysed by ^1^H NMR (AVANCE 400MHZ).

### 2.4 Drug encapsulation efficiency (EE)

The amount of SeMC was measured using the absorbance of SeMC NPs at 570 nm according to the requirement of the ninhydrin chromogenesis method for the determination of amino acid content.^37^ The EE of SeMCs was calculated according to the following equations: EE (%) = (*M*_2_/*M*_1_) × 100, where *M*_1_ is the initial weight of SeMC, *M*_2_ is the weight of SeMC in NPs.

### 2.5 In vitro cytotoxicity assay

Cells were cultured in 96‐well plates at a density of 1 × 10^4^ cells per well and cultured for 12 h at 37°C. After incubation with SeMC NPs for 48 h, the cells were incubated with fresh media containing 10% CCK‐8 solution for 30 min at 37°C. The viabilities of the cells were determined by measuring the absorbance at 450 nm with a microplate reader.

### 2.6 In vitro ROS detection

L‐02 cells (2 × 10^5^ cells per well) were cultured in 24‐well plates overnight. After incubation with SeMCs or SeMC NPs for 24 h, cells were washed with PBS and then incubate with APAP or CCl_4_ for 12 h. After that, the cells were washed twice with PBS and incubated with RPMI 1640 containing 20 μM DCFH‐DA for 40 min. Fluorescence imaging was performed on a Leica TCS SP8 confocal laser scanning microscope (Ex = 488 nm, Em = 520 nm).

### 2.7 In vitro cellular uptake

The hepatocyte‐targeting ability of GA‐SeMC NPs was evaluated with an in vitro cellular uptake assay. Rhodamine B was used as the fluorescent dye and observed by confocal laser scanning microscopy (CLSM; TCS SP8; Leica Microsystems). First, L‐02 cells harvested in the exponential growth phase were seeded in 24‐well plates at a density of 2 × 10^5^ cells/well and incubated overnight at 37°C. Subsequently, the cells were incubated with fresh RPMI 1640 containing RhB‐labelled SeMC NPs or RhB‐labelled GA‐ SeMC NPs for 1 or 1.5 h at 37°C. The cells were then washed three times with PBS and fixed with a 4% paraformaldehyde for 10 min at 25°C. Finally, the cells were observed by CLSM.

### 2.8 Animal treatment

Female BALB/c mice (6–8 weeks) were purchased from SLAC Laboratory Animal Co. Ltd. (Shanghai, China). All mouse experiments were carried out according to protocols approved by the Animal Care and Use Committee of Shanghai Jiao Tong University School of Medicine (A‐2022‐115). To explore the protective effect of GA‐SeMC NPs in ameliorating APAP‐ or CCl_4_‐induced liver injury, mice were intravenously injected with GA‐SeMC NPs every other day for a total of three doses before APAP/CCl_4_ treatment. The extent of liver injury was then determined by measuring serum levels of ALT and AST at 24 h after APAP (300 mg kg^−1^) or CCl_4_ (10 mL kg^−1^) administration. The capability of GA‐SeMC NPs to ameliorate APAP‐ or CCl_4_‐induced liver injury was compared to that of unmodified SeMC NPs and free SeMCs.

The mice were randomly divided into five groups (six mice per group). After the mice were anaesthetised, blood and liver samples were collected for further experiments.

### 2.9 Detection of ALT, AST and LDH activities

L‐02 cells were cultured in 12‐well plates at a density of 5 × 10^5^ cells per well and cultured overnight. After incubation with SeMCs, PNPs and SeMC NPs for 24 h, the cells were washed with PBS, and then treated with APAP (10 mM) or CCl_4_ (40 mM) for 24 h, respectively. The supernatants of the cells were collected according to alanine aminotransferase (ALT, C009‐2‐1), aspartate aminotransferase (AST, C010‐2‐1), lactate dehydrogenase (LDH, A020‐2‐2) kit instruction (Nanjing Jiancheng Bioengineering Institute), the activities of ALT, AST, LDH in cell supernatant were detected.

To determine liver injury in mice, ALT, AST, LDH activities were determined using the commercial assay kits from Nanjing Jiancheng Bioengineering Institute (Nanjing, China), according to the manufacturer's instructions.

### 2.10 Detection of SOD, GSH‐Px and MDA activities

The liver homogenate was prepared, and the hepatic protein content was determined as described previously. The supernatant was used to determine the superoxide dismutase (SOD, A001‐3) activity, glutathione peroxidase (GSH‐Px, A005‐1) activity and malondialdehyde (MDA, A003‐1) content using commercial kits from Nanjing Jiancheng Bioengineering Institute, according to the manufacturer's instructions.

### 2.11 Histopathology analysis

Fresh liver tissues from the same lobes were collected, trimmed to a thickness of about 2 mm, and then fixed in 10% buffered formaldehyde solution. Fixed tissues were cut into sections of 2 μm and stained using hematoxylin and eosin (H&E) followed by examination under a light microscope.

### 2.12 Statistical Analysis

Statistical analyses were performed with the GraphPad Prism. All data were representative of ≥3 independent experiments and presented as mean ± SD or mean ± SEM. Student's *t* test was used to determine the statistical significance of the differences between two groups. ns means not significance, *p < 0.05, **p < 0.01, ***p < 0.001.

## 3 RESULTS

### 3.1 Fabrication and characterization of SeMC NPs

**FIGURE 1:**
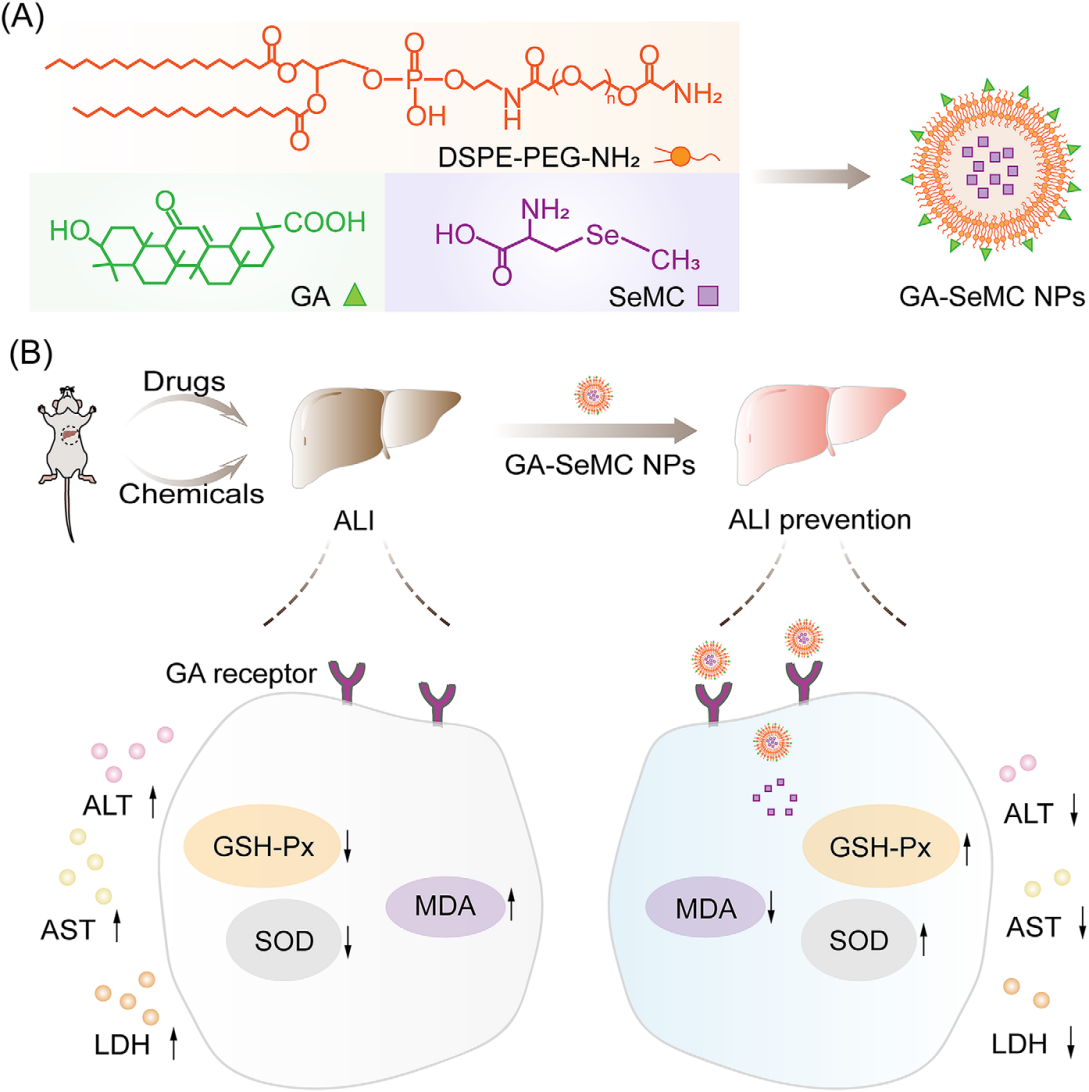
Fabrication of hepatocyte‐targeted nano‐antioxidants for the treatment of ALI. (A) Schematic diagram for the composition of GA‐SeMC NPs. (B) Schematic illustration of the hepatoprotective effects of GA‐SeMC NPs in ALI mouse models.

GA‐SeMC NPs were synthesized via a two‐step procedure. First, GA was covalently conjugated with the amino terminal of DSPE‐PEG‐NH_2_ and form DSPE‐PEG‐GA via EDC/NHS coupling chemistry. Then, GA‐SeMC NPs composed of unmodified DSPE‐PEG‐NH_2_, DSPE‐PEG‐GA and SeMC were obtained by a thin‐film hydration method (Figure 1A). After intravenous injection, we expected that the GA‐SeMC NPs could efficiently accumulate in the liver tissue via binding to the GA receptors on the hepatocyte surface and exhibit protective effects on the drug‐ or chemical‐induced ALI mouse models, including the elevation of endogenous antioxidant enzyme expression, suppression of the hepatic oxidative stress, and restoration of liver functions (Figure 1B).

**FIGURE 2:**
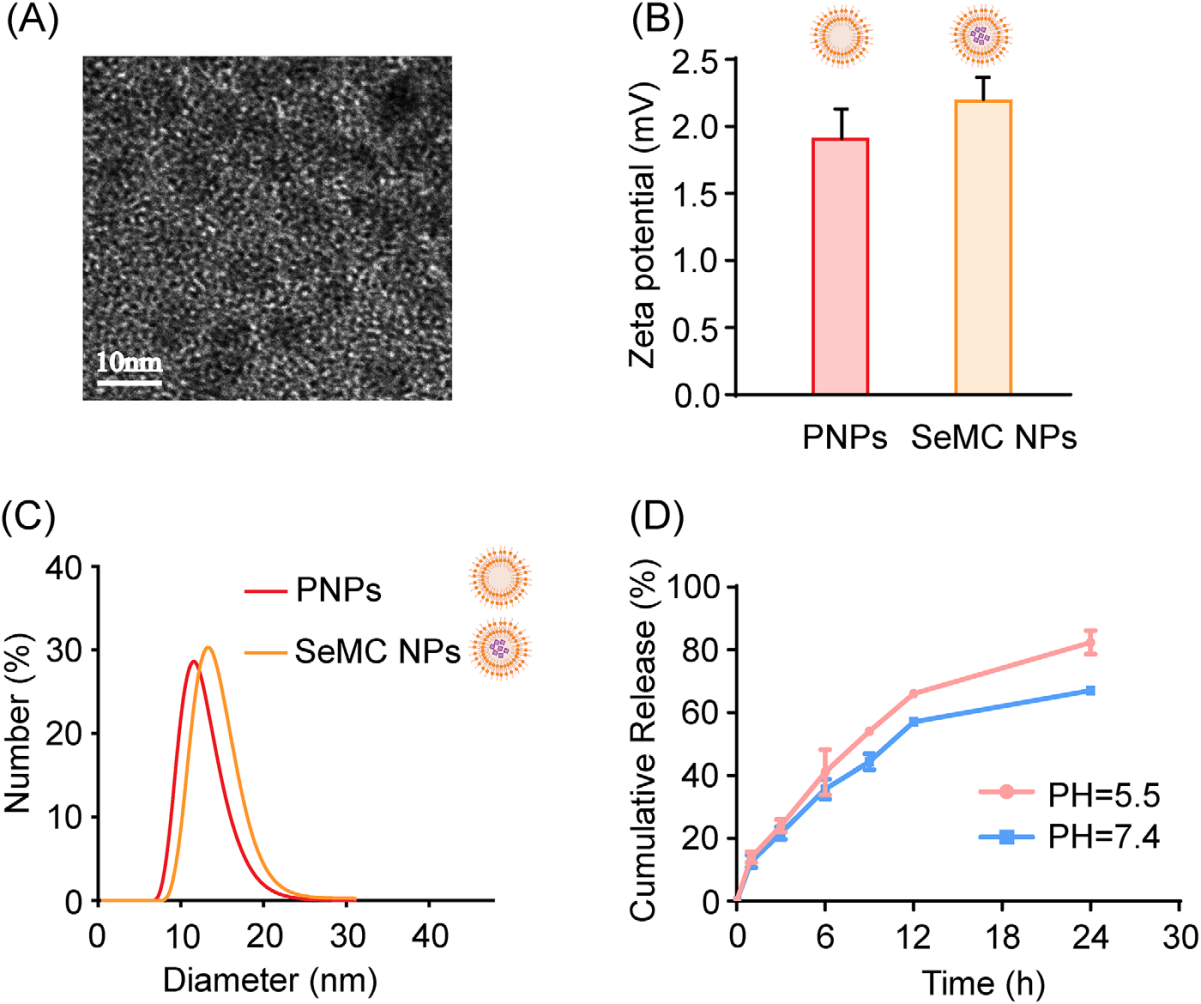
Characterization of SeMC NPs. (A) Transmission electron microscope (TEM) image of the SeMC NPs. (B) The zeta potential of unloaded polymeric nanoparticles (PNPs) and SeMC NPs. (C) The dynamic light scattering (DLS) analysis of PNPs and SeMC NPs. (D) The drug release characteristics of the SeMC NPs. Data are represented as mean ± SD (*n* = 3).

In a pilot study, different kinds of amphiphilic polymers were tested to encapsulate SeMCs and form desirable SeMC NPs. The particle sizes, zeta potentials and the SeMCs encapsulation efficiencies of different kinds SeMC NPs were studied. As shown in Table S1, the SeMC NPs composed of PCL‐PEG or PCL‐PEI molecules presented relative larger sizes and lower SeMC encapsulation efficiencies, while those composed of DSPE‐PEG‐OH or DSPE‐PEG‐NH_2_ molecules showed smaller sizes and higher SeMC encapsulation efficiencies. In addition, the SeMC NPs composed of DSPE‐PEG‐OH and the SeMC NPs composed of DSPE‐PEG‐NH_2_ had similar particle sizes and SeMC encapsulation efficiencies. Compared to the negatively charged SeMC NPs composed of DSPE‐PEG‐OH molecules (−9.69 ± 0.5 mV), the SeMC NPs composed of DSPE‐PEG‐NH_2_ were positively charged (1.6 ± 0.4 mV). Given that the cellular uptake efficiency of negatively charged NPs is lower than that of positively charged NPs, and the NPs with amino terminus are more convenient to covalently link with targeting molecules of hydroxyl terminus, SeMC NPs composed of DSPE‐PEG‐NH_2_ were chosen for the subsequent experiments. More comprehensive characterization of the SeMC NPs composed of DSPE‐PEG‐NH_2_ (referred to as SeMC NPs hereafter) were performed and the data were shown in Figure 2 and Figure S1. The SeMC NPs showed a spherical shape and uniform size of 10.15 ± 2.9 nm under the transmission electron microscope (TEM) (Figure 2A and Figure S1A). The SeMC NPs and unloaded DSPE‐PEG‐NH_2_ polymeric nanoparticles (PNPs) showed comparable positive surface potentials. After the loading of SeMCs, the sizes of NPs were slightly increased (Figure 2B,C). A period of five‐week stability study showed no significant changes in particle size for the SeMC NPs, showing a decent stability (Figure S1B).

To investigate the drug release characteristics of the SeMC NPs, the release profile of SeMCs from NPs at different pH values was evaluated using the dialysis method. As shown in Figure 2D, in both cytoplasm‐mimicking environment (pH 7.4) and the acidic endosome‐mimicking environment (pH 5.5), SeMC NPs presented a fast releasing behaviour in the first 6 h, with the releasing levels of ~36% at pH 7.4 and ~41% at pH 5.5. Compared to the cytoplasmic environment, SeMC NPs had a slightly faster releasing rate under acidic condition, and the cumulative releasing levels reached approximately 82% in 24 h. Moreover, the biocompatibility of the synthesized SeMC NPs were evaluated by examining the cytotoxicity of these NPs in the cultured cells. Figure S2 exhibited that SeMC NPs had a negligible influence on the viabilities of L‐02 normal liver cells at various SeMC concentrations (50–250 μg mL^−1^) within 48 h, indicating good biocompatibility of SeMC NPs.

### 3.2 Hepatoprotective effects of SeMC NPs in vitro

**FIGURE 3:**
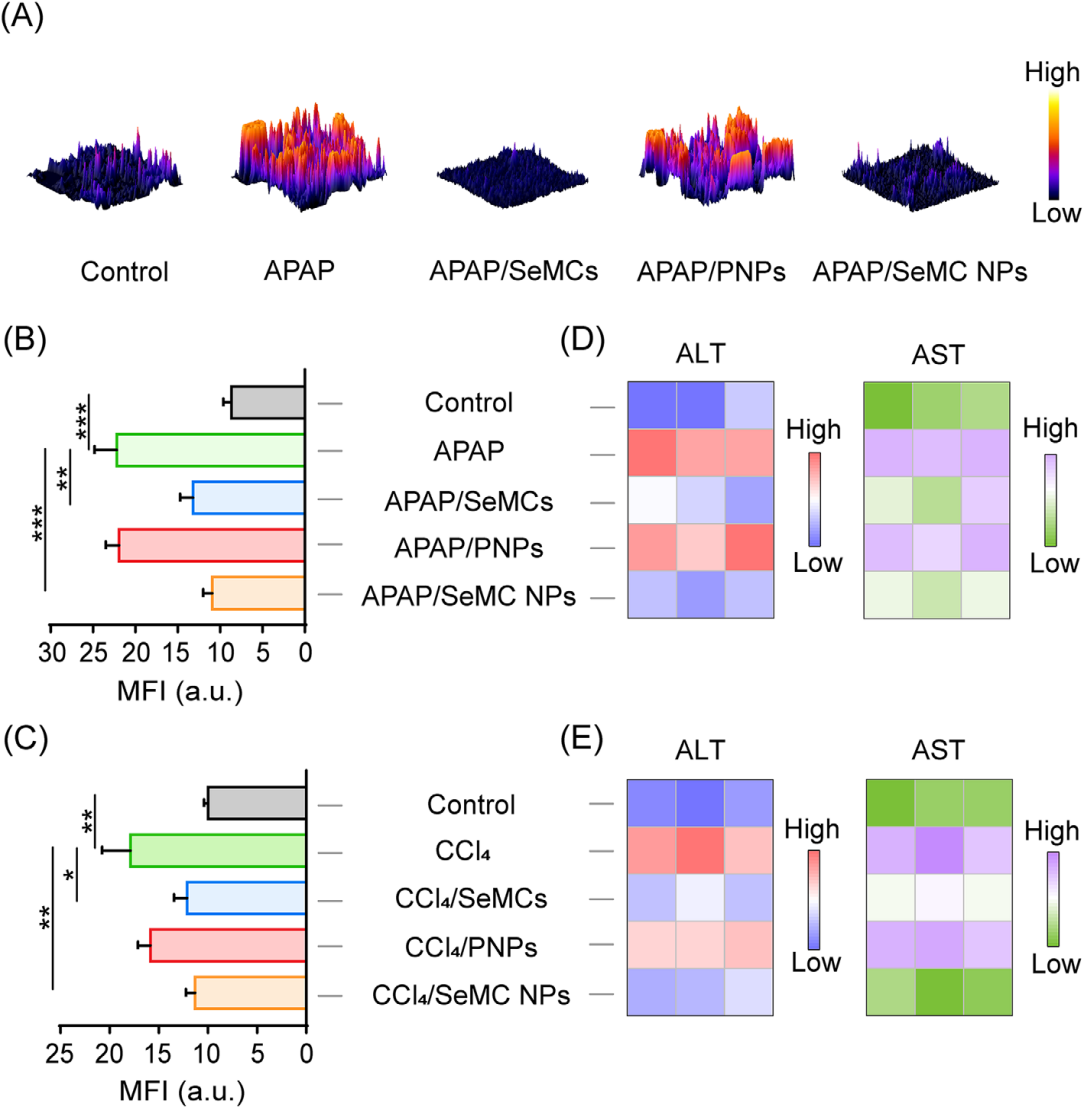
Hepatoprotective effects of SeMC NPs. (A) 2.5D images of the intracellular ROS levels in L‐02 cells with indicated treatments detected by the DCFH‐DA probe. (B, C) The geometric mean fluorescence intensities (MFI) of ROS in L‐02 cells with indicated treatments assessed with the Leica LAS AF Lite software. Data are represented as mean ± SD (*n* = 10). *p < 0.05, **p < 0.01 and ***p < 0.001. See also Figures S4 and S5 for representative fluorescence images. (D, E) Colour coded grid showing ALT and AST values in L‐02 cells with indicated treatments. Colours ranging from dark to light represent the lowest and the highest intensity. Data are represented as mean ± SD (*n* = 6).

Two in vitro models of acute hepatocellular injury were constructed by administering acetaminophen (APAP) or carbon tetrachloride (CCl_4_) to L‐02 cells. The secreted levels of alanine aminotransferase (ALT), aspartate aminotransferase (AST) and lactate dehydrogenase (LDH) are important indicators to evaluate the degree of hepatocyte injury.^38^, ^39^ Figure S3 showed that all these parameters in L‐02 cells administrated with 10 mM APAP or 40 mM CCl_4_ were obviously higher than those of PBS treated cells, demonstrating that different experimental models of acute hepatocellular injury were successfully constructed. Considering oxidative stress induced by excessive ROS is the main mechanism underlying acute liver injury, the antioxidative properties of SeMC NPs in the injured L‐02 cells were investigated firstly. The intracellular ROS was visualized and quantified by the 2′,7′‐dichlorofluorescein (DCF, with green fluorescence), the oxidative product of 2′,7′‐dichlorodihydrofluoresceindiacetate (DCFH‐DA). As expected, the green fluorescence signals presented by the confocal images were obviously increased in APAP‐challenged L‐02 cells compared to those normal cells, demonstrating that APAP indeed induced intracellular ROS accumulation. As shown in Figure 3A and Figure S4, compared with PBS or PNPs administration group, the ROS levels in APAP‐challenged L‐02 cells decreased significantly in the SeMCs or SeMC NPs treatment group. Moreover, SeMC NPs and SeMCs exhibited comparable efficacy in ROS elimination, indicating that SeMCs possessed potent antioxidant capacity and the nanoformulation procedure of SeMCs did not affect this capacity. The corresponding statistical analyses were also displayed in Figure 3B, which quantitatively showed the same tendency as that in confocal images. Whereafter, the antioxidant activities of the SeMC NPs were further verified in CCl_4_‐treated L‐02 cells. Exposure to CCl_4_ led to an accumulation of the intracellular ROS, while both free SeMCs and SeMC NPs treatment repressed the intracellular ROS accumulation induced by CCl_4_ in L‐02 cells (Figure 3C and Figure S5).

To further confirm the hepatocellular protective effects of the SeMC NPs in APAP‐induced hepatocellular injury model, we measured the levels of ALT and AST, two important indexes of hepatocyte injury. As expected, the levels of ALT and AST released from L‐02 cells noticeably increased after APAP‐challenging and the administration of SeMC NPs obviously reduced both indexes, indicating the restoration of hepatocyte functions (Figure 3D and Figure S6A,B). In the meanwhile, the unloaded PNPs showed no effects on hepatocellular protection. Similarly, hepatocellular protective efficacy of SeMC NPs was also verified in CCl_4_‐induced hepatocellular injury model. Pretreating L‐02 cells with SeMC NPs for 24 h before CCl_4_ treatment notably decreased the levels of secreted ALT and AST compared to CCl_4_ treatment alone (Figure 3E and Figure S6C,D). Apart from ALT and AST, lactate dehydrogenase (LDH) is another index of hepatocyte function that is released during cell injury. Like the other two indexes, the uptake of SeMC NPs obviously reduced the levels of secreted LDH in the above‐mentioned two experimental models of acute hepatocellular injury (Figure S7). Moreover, the protective effects of SeMC NPs towards hepatocytes under various stimulations were further verified by the CCK8 assays. As shown in the Figure S8, SeMC NPs significantly ameliorated the decrease of cell viabilities caused by APAP or CCl_4_, further verifying the protective effects of SeMC NPs against hepatocyte injury.

### 3.3 Fabrication and characterization of hepatocyte‐targeting GA‐SeMC NPs

**FIGURE 4:**
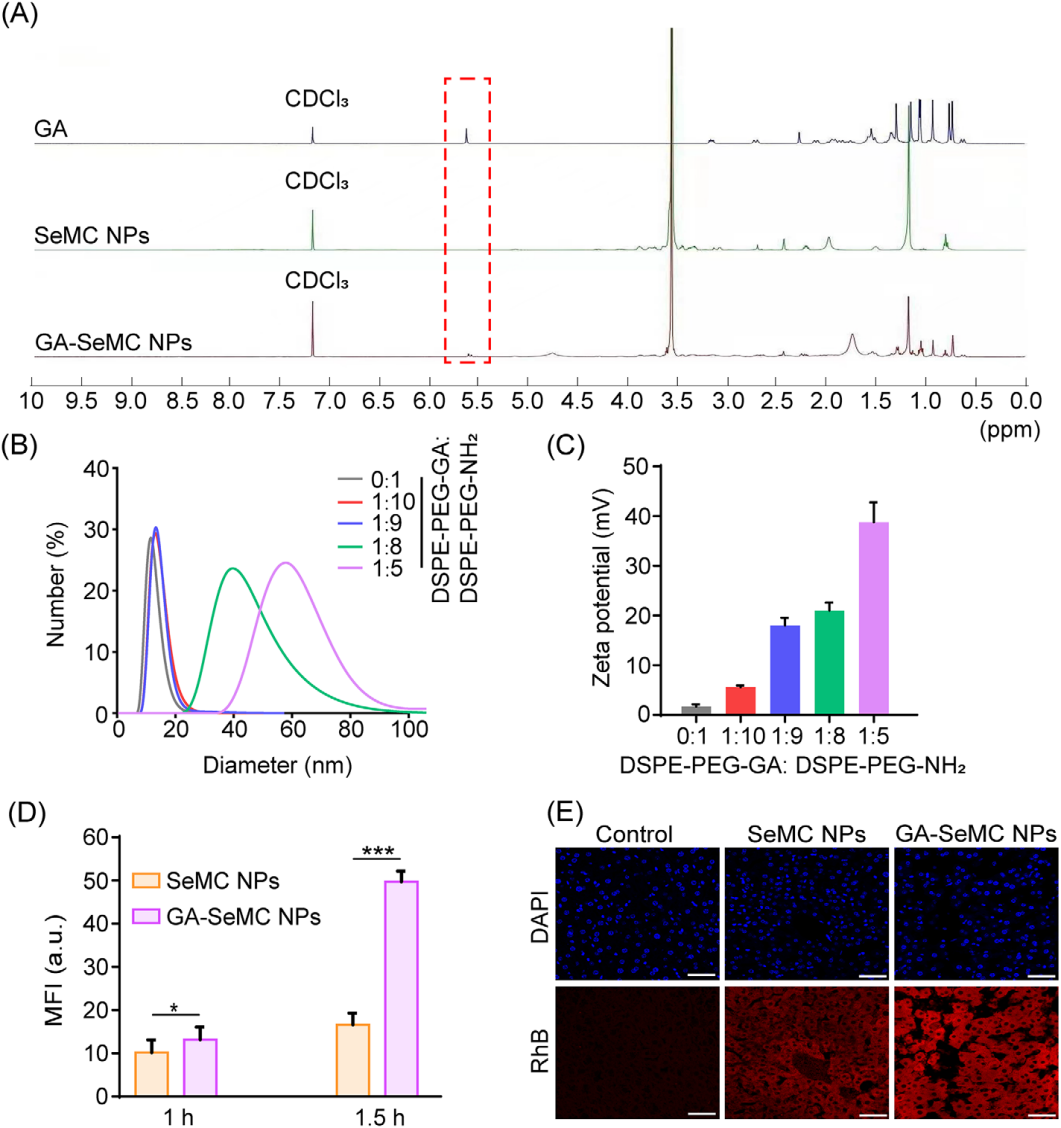
Fabrication and characterization of hepatic targeting SeMC NPs. (A) ^1^H NMR spectra of GA, SeMC NPs and GA‐SeMC NPs. (B) The DLS analysis of SeMC NPs with different GA content. (C) The zeta potential of SeMC NPs with different GA content. (D) Uptake of RhB‐labelled SeMC NPs or GA‐SeMC NPs in L‐02 cells. See also Figure S9A for the representative fluorescence images. Data are represented as mean ± SD (*n* = 10). *p < 0.05, ***p < 0.001. (E) The accumulation of RhB‐labelled SeMC NPs or GA‐SeMC NPs in liver. Scale bars: 75 μm.

To endow the SeMC NPs with targeting ability for hepatocytes, we functionalized them with GA, a well‐established liver targeting ligand,^40^, ^41^ and fabricated GA‐SeMC NPs. The ^1^H NMR spectra of GA, SeMC NPs, and GA‐SeMC NPs were shown in Figure 4A. In comparison to unmodified SeMC NPs, the additional signal peak at ~5.7 ppm that attributed to the protons of olefinic bond (—(C=O)—CH=C—) in GA was detected in GA‐SeMC NPs, demonstrating the conjugation of GA to the GA‐SeMC NPs.^42^, ^43^ To optimize the particle sizes and drug encapsulation efficiencies of the GA‐SeMC NPs, different ratios of DSPE‐PEG‐GA to DSPE‐PEG‐NH_2_ during the GA‐SeMC NP preparation were tested. Figure 4B,C presented the changes in the particle sizes and zeta potentials of GA‐SeMC NPs with the increases of DSPE‐PEG‐GA to DSPE‐PEG‐NH_2_ molar ratios. As indicated in Figure 4B, the molar ratios of DSPE‐PEG‐GA to DSPE‐PEG‐NH_2_ could affect the sizes of GA‐SeMC NPs. Notably, no obvious changes in the particle size of GA‐SeMC NPs were observed at the molar ratios of 1:10 and 1:9 in comparison to SeMC NPs. A significant increase in the size of GA‐SeMC NPs was detected when the molar ratio of DSPE‐PEG‐GA to DSPE‐PEG‐NH_2_ was further increased. This might be caused by the increased hydrophobicity of DSPE‐PEG‐NH_2_ polymer molecules following the modification with hydrophobic GA molecules. When the molar ratios of DSPE‐PEG‐GA to DSPE‐PEG‐NH2 were low (e.g., 1:10 or 1:9) in GA‐SeMC NPs, the aforementioned increase in hydrophobicity was insufficient to alter the self‐assembly behaviour of the mixed polymers. As the proportion of DSPE‐PEG‐GA gradually increases, this GA‐mediated increase in hydrophobicity could affect the self‐assembly behaviour of the mixed polymers, ultimately influencing the size of the micellar nanoparticles. According to the drug encapsulation efficiency tests, no statistical differences in SeMC encapsulation efficiency of GA‐SeMC NPs were observed at the molar ratios of 1:10 and 1:9 in comparison to SeMC NPs. In addition, the zeta potentials of the GA‐SeMC NPs increased with the increase of molar ratios of DSPE‐PEG‐GA to DSPE‐PEG‐NH_2_ (Figure 4C). Thus, after a comprehensive consideration, GA‐SeMC NPs with a 1:9 molar ratio of DSPE‐PEG‐GA to DSPE‐PEG‐NH_2_ were mainly utilized for the following experiments.

The targeting ability of GA‐SeMC NPs for hepatocytes was monitored by fluorescence imaging. As shown in Figure 4D and Figure S9A, the L‐02 cells treated with RhB‐labelled SeMC NPs showed much weaker intensities of the fluorescence comparing to the L‐02 cells treated with RhB‐labelled GA‐SeMC NPs, reflecting the enhanced efficiency in the hepatocyte‐targeting ability of GA‐SeMC NPs. To verify the liver‐targeting effects of GA‐SeMC NPs in vivo, BALB/c mice were injected intravenously with RhB‐labelled GA‐SeMC NPs or RhB‐labelled SeMC NPs. As shown in Figure 4E and Figure S9B, the animals were sacrificed 6 h after drug administration, and liver tissues were harvested and sectioned to monitor the fluorescence signal of RhB by confocal laser scanning microscopy. Compared to RhB‐labelled SeMC NPs, RhB‐labelled GA‐SeMC NPs were accumulated at higher levels in the liver, with strong fluorescence detected in liver tissue slices 6 h after injection, indicating that the effective liver‐targeting ability of GA‐SeMC NPs in vivo.

### 3.4 Protective effects of GA‐SeMC NPs on ALI mice

**FIGURE 5:**
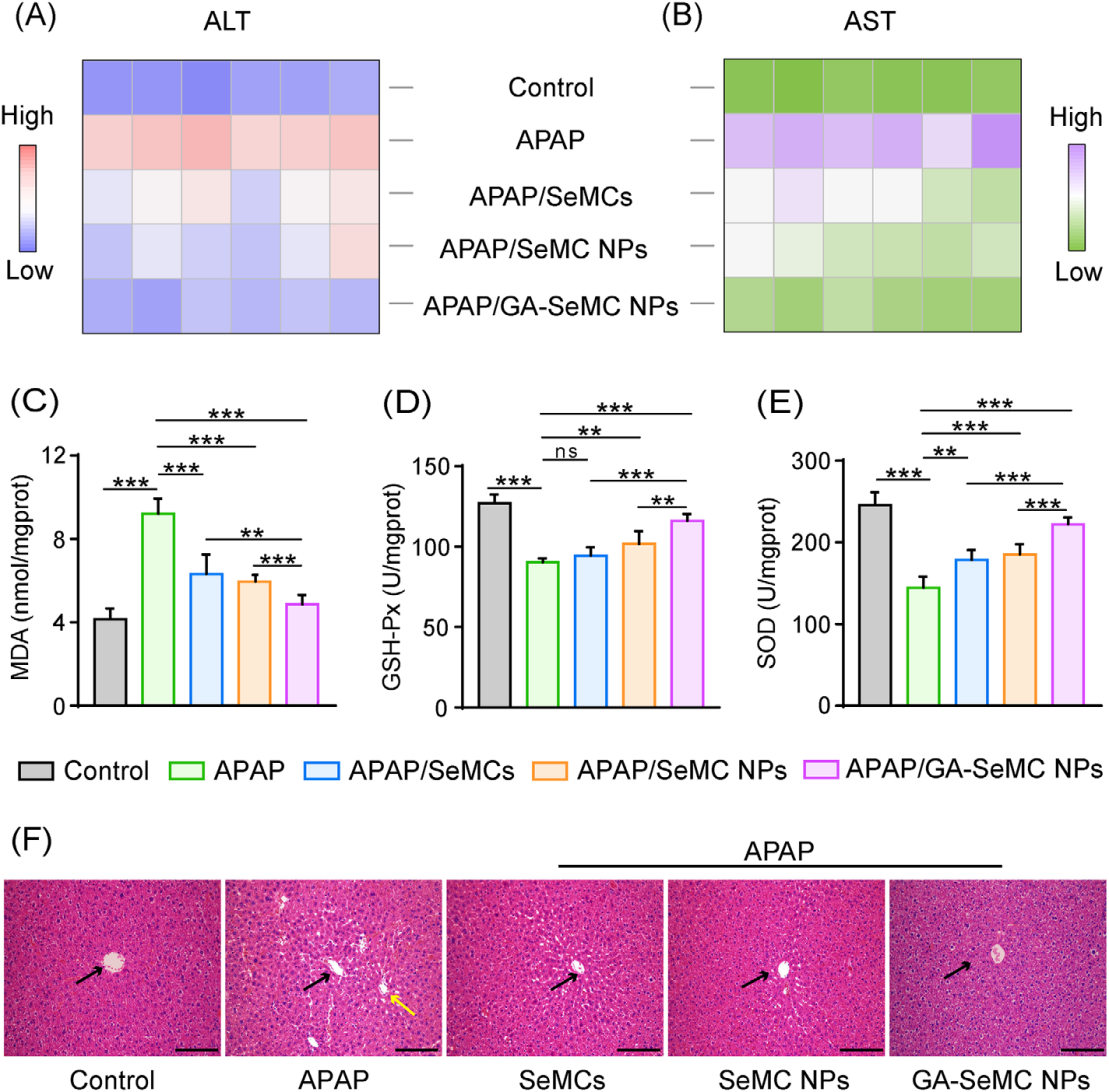
SeMC NPs attenuate APAP‐induced ALI in mice. (A, B) Colour coded grid showing serum ALT and AST levels in mice with different treatments. Colours ranging from orange to blue represent the lowest and the highest intensity. Complete data are provided in Figure S11. Data are represented as mean ± SD (*n* = 6). (C–E) The hepatic MDA (C), GSH‐Px (D) and SOD (E) levels in mice with different treatments. Data are represented as mean ± SD (*n* = 6). *p < 0.05, **p < 0.01, ns means not significant. (F) Hematoxylin and eosin (H&E) staining of the liver tissues collected from mice with different treatments. Black arrows indicate the central vein. Yellow arrow indicates hepatic cell necrosis and vacuolization. Scale bar: 100 μm.

Based on the superior antioxidant activities and the satisfying hepatocellular protective effects of SeMC NPs in vitro, two mouse models of ALI induced by APAP or CCl_4_ were established to evaluate the in vivo hepatoprotective effects of GA‐SeMC NPs. To establish a suitable mouse model of APAP‐induced ALI,^44^, ^45^ the mice were intraperitoneally injected with APAP (300 mg kg^−1^), and the serum levels of ALT and AST were measured as the indexes of liver injury. As indicated in Figure S10, the serum levels ALT and AST in the mice were obviously increased 12 h after the injection of APAP, indicating successful establishment of APAP‐induced ALI model. To explore the protective effect of GA‐SeMC NPs in ameliorating APAP‐induced liver injury, the mice were intravenously injected with GA‐SeMC NPs every other day for a total of three doses before APAP treatment. The extent of liver injury was then determined by measuring serum levels of ALT and AST 24 h after APAP administration. The capability of GA‐SeMC NPs to ameliorate APAP‐induced liver injury was compared to that of unmodified SeMC NPs and free SeMCs. Compared with the control group, serum levels of ALT and AST of the mice in APAP‐challenged group were significantly increased. Among the APAP‐treated groups, the mice pretreated with the GA‐SeMC NPs presented significantly reduced ALT and AST levels. In comparison, free SeMCs or SeMC NPs with equivalent SeMC doses to that in GA‐SeMC NPs presented moderate effects in reducing serum ALT and AST levels. Moreover, GA‐SeMC NPs treatment showed a larger protective benefit than the free SeMCs or SeMC NPs group, as evidenced by serum ALT and AST levels in the GA‐SeMC NPs treatment group being closer to those in the normal control group (Figure 5A,B and Figure S11).

As oxidative stress is a critical factor in APAP‐induced hepatotoxicity, the hepatic oxidative stress in liver tissue was evaluated by measuring the levels of malondialdehyde (MDA), an end product of lipid peroxidation that could reflect the levels of cellular oxidative stress.^46^ Compared with the untreated control group, APAP treatment significantly increased the hepatic MDA levels, suggesting that severe oxidative stress was induced by APAP. As shown in Figure 5C, free SeMCs, SeMC NPs or GA‐SeMC NPs treatments reduced the MDA accumulation induced by APAP in the liver. GA‐SeMC NPs repressed MDA accumulation more efficiently than free SeMCs and SeMC NPs. GSH‐Px and superoxide dismutase (SOD) are two important antioxidant enzymes, which protect the organisms from oxidative damage.^46^, ^47^, ^48^ While APAP intoxication caused significant reduction of the activities of hepatic GSH‐Px and SOD, either free SeMCs, SeMC NPs or GA‐SeMC NPs ameliorate this effect and restore GSH‐Px and SOD activities (Figure 5D,E). Of note, as GA‐SeMC NPs possessed the capacity of hepatic targeting, they presented superior effects to free SeMCs and SeMC NPs on hepatoprotection against APAP‐induce damage. The histopathological analysis further supported the excellent protection of APAP‐induced hepatotoxicity by GA‐SeMC NPs. Representative images of hematoxylin–eosin (H&E) staining of liver tissues in the groups with different treatment were showed in Figure 5F. Compared to the PBS‐treated control group, severe hepatic lesions were observed in APAP‐treated group, showing hepatic cell necrosis and vacuolization. Administration of SeMCs or SeMC NPs resulted in moderate improvement in liver histological lesions, while the mice injected with GA‐SeMC NPs showed liver histological characteristics similar to those of normal healthy mice.

**FIGURE 6:**
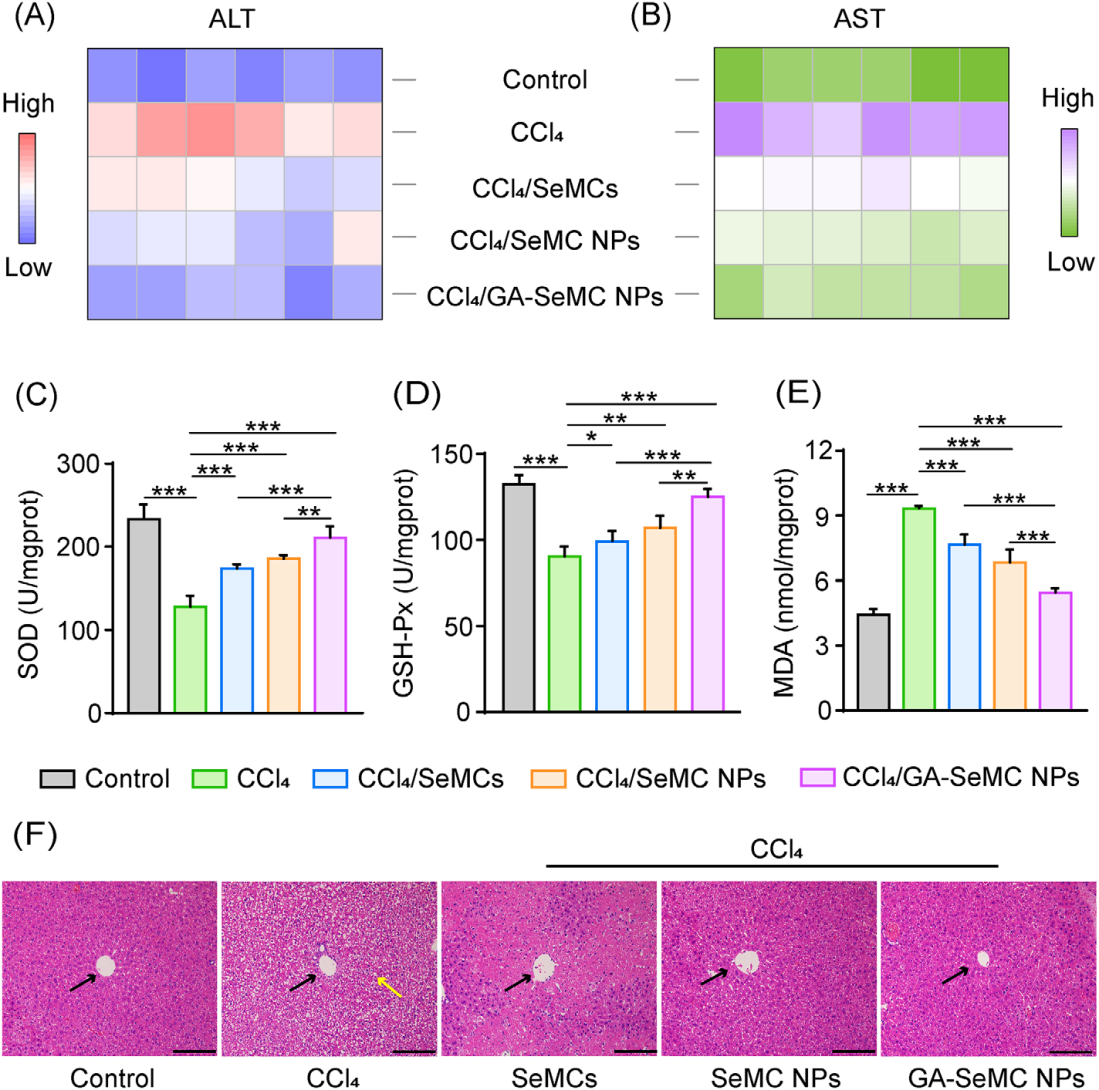
SeMC NPs relieve CCl_4_‐induced ALI in mice. (A, B) Colour coded grid showing serum ALT and AST levels in mice with different treatments. Colours ranging from blue to red represent the lowest and the highest intensity. Complete data are provided in Figure S13. Data are represented as mean ± SD (*n* = 6). (C–E) The hepatic SOD (C), GSH‐Px (D) and MDA (E) levels in mice with different treatments. Data are represented as mean ± SD (*n* = 6). *p < 0.05, **p < 0.01. (F) H&E staining of the liver tissues collected from mice with different treatments. Black arrows indicate the central vein. Yellow arrow indicates hepatic cell necrosis and vacuolization. Scale bar: 100 μm.

To further verify the hepatoprotective effects of the GA‐SeMC NPs, the mouse model of CCl_4_‐induced ALI was used (Figure S12). As expected, the intraperitoneal injected CCl_4_ led to significant elevation in the serum levels of ALT and AST, whereas pretreatment of GA‐SeMC NPs markedly inhibited the elevation of these two indexes induced by CCl_4_ (Figure 6A,B and Figure S13). Comparing to the normal control mice, the levels of antioxidant enzymes SOD and GSH‐Px were significantly decreased in CCl_4_ injected mice, while the oxidative product MDA was markedly increased. In the mice pretreated with GA‐SeMC NPs before CCl_4_ injury, the levels of SOD, GSH‐Px and MDA were all significantly reversed, showing the superior hepatoprotective effects of GA‐SeMC NPs (Figure 6C–E). Histopathological analysis of liver tissues further demonstrated the hepatoprotective effect of GA‐SeMC NPs on CCl_4_‐induced liver injury (Figure 6F).

## 4 CONCLUSIONS

In the present study, we developed a new strategy against ALI by encapsulating SeMC, a new generation of antioxidant organic Se compound, into hepatocyte‐targeting amphiphilic micellar NPs, which exhibited a promising hepatoprotective effect on the mouse models of ALI. At the cellular level, by efficiently scavenging the excessive intracellular ROS, nanoformulated SeMCs could significantly ameliorate the hepatocyte injury indexes and the decrease of cell viability caused by APAP or CCl_4_. The in vivo studies revealed that treatment with nanoformulated SeMCs could effectively prevent ALI caused by APAP or CCl_4_ and achieve normal liver functions and restore the levels of endogenous antioxidant enzymes. It is worth noting that the present study solely focused on the effectiveness of nanoformulated SeMCs in preventing potential ALI. Further studies are required to explore whether nanoformulated SeMCs are effective in mitigating the progression of ALI after it has already occurred. Taken together, nanoformulated SeMCs exhibited a satisfying protective effect against liver intoxication, offering a promising potential solution for the prevention of ALI and other hepatic diseases.

## AUTHOR CONTRIBUTIONS

The manuscript was written through contributions of all authors. All authors have given approval to the final version of the manuscript.

## CONFLICT OF INTEREST STATEMENT

The authors declare no conflict of interest.

## Supporting information


**Data S1.** Supporting Information

## Data Availability

The data that support the findings of this study are available from the cor‐responding author upon reasonable request.
