# Supplementary material for: Preventing acute liver injury via hepatocyte‐targeting nano‐antioxidants
Source: Cell Prolif. 2023 May 4;56(12):e13494. doi: 10.1111/cpr.13494 (PMC10693184; doi:10.1111/cpr.13494)
Supplement: Supplementary file 1 — Data S1. Supporting Information [file CPR-56-e13494-s001.pdf]

Supporting Information

Preventing Acute Liver Injury via Hepatocyte-Targeting Nano-Antioxidants

Xuejiao Yuan[1,‡], Yanfeng Zhou[2,‡], Jinli Sun[2], Shanshan Wang[1], Xingjie Hu[2], Jiyu Li[2,3], Jing Huang,[4,*] Nan Chen[1,*]

[1] College of Chemistry and Materials Science, The Education Ministry Key Lab of Resource Chemistry, Joint International Research Laboratory of Resource Chemistry of Ministry of Education, Shanghai Key Laboratory of Rare Earth Functional Materials, and Shanghai Frontiers Science Center of Biomimetic Catalysis, Shanghai Normal University, Shanghai 200234, China

[2] School of Public Health, Shanghai Jiao Tong University School of Medicine, Shanghai 200025, China

[3] He'nan Xibaikang Health Industry Co., Ltd, Jiyuan 454650, China

[4] Department of Neurology, Xuhui District Central Hospital, Shanghai 200032, China

*Correspondence: Nan Chen, nchen@shnu.edu.cn;

Jing Huang, huangjing.doc@hotmail.com

‡ These authors contributed equally to this work.

Sample	Particle size (nm)	Zeta potential (mV)	Encapsulation efficiency (%)
DSPE-PEG_{2000}	16.6 ± 0.2	-8.20 ± 0.2	22.2 ~ 35.0
DSPE-PEG_{2000}-OH	18.5 ± 0.2	-9.69 ± 0.5	21.1 ~ 36.0
DSPE-PEG_{2000}-NH_2	15.5 ± 0.4	1.60 ± 0.4	27.1 ~ 32.0
PCL_{1000}-PEG_{2000}-NH_2	68.06 ± 0.3	16.3 ± 0.3	20.0 ~ 24.3
PCL_{2000}-PEG_{5000}-NH_2	255.0 ± 0.4	-5.06 ± 0.2	19.9 ~ 22.4
PCL_{5000}-PEI_{2000}	614.9 ± 0.3	50.8 ± 0.4	2.18 ~ 5.06

Table S1 The particle sizes, zeta potentials and the SeMC encapsulation efficiencies of different kinds of SeMC NPs. Data are represented as mean ± SD (n = 3).

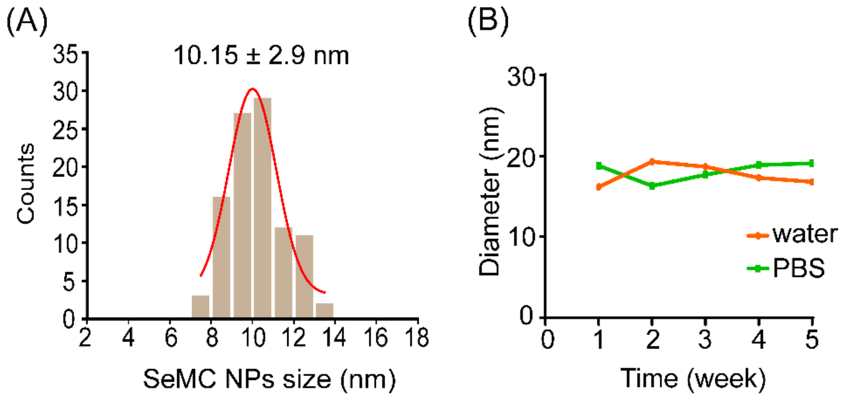

Figure S1 Characterization of SeMC NPs. (A) The corresponding size distribution of the SeMC NPs. (B) Hydrodynamic diameters of SeMC NPs at different time points were measured. Data are represented as mean ± SD (n = 3).

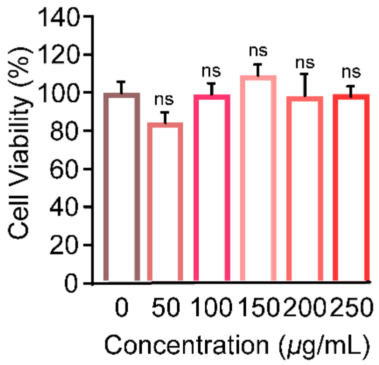

Figure S2 Cell viabilities of L-02 cells exposure to SeMC NPs for 48 hours. Data are represented as mean ± SD (n = 3). ns means not significant.

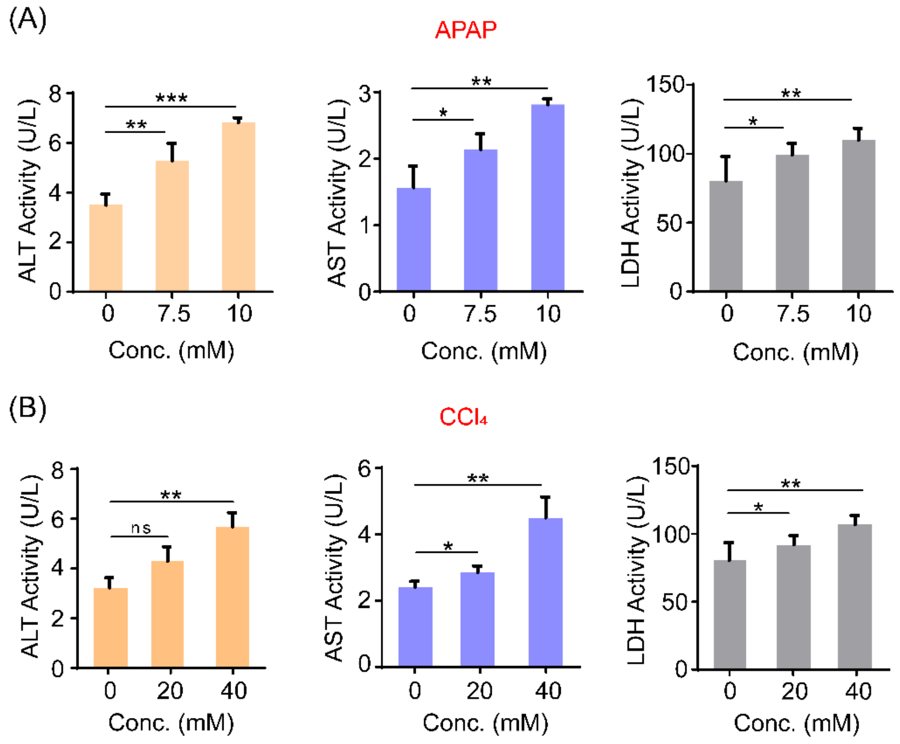

Figure S3 Biochemical analysis of L-02 cell supernatants. (A-B) The levels of ALT, AST and LDH in the L-02 cell supernatant after treatments with chemicals of indicated concentrations. Data are represented as mean ± SD (n = 3). *$p < 0.05$, **$p < 0.01$, and ***$p < 0.001$.

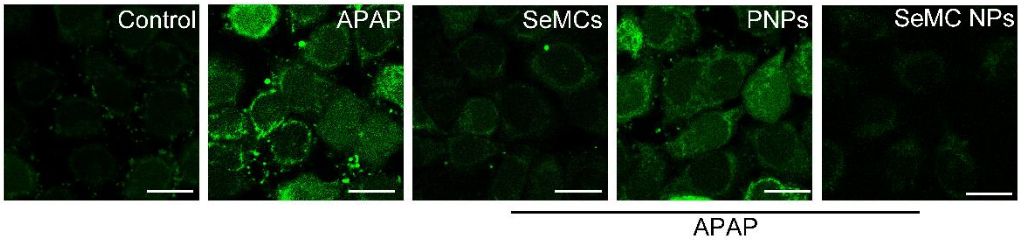

Figure S4 Effects of SeMC NPs on APAP-treated L-02 cells. Intracellular ROS levels in L-02 cells with indicated treatments detected by the DCFH-DA probe. Scale bar: 10 μm.

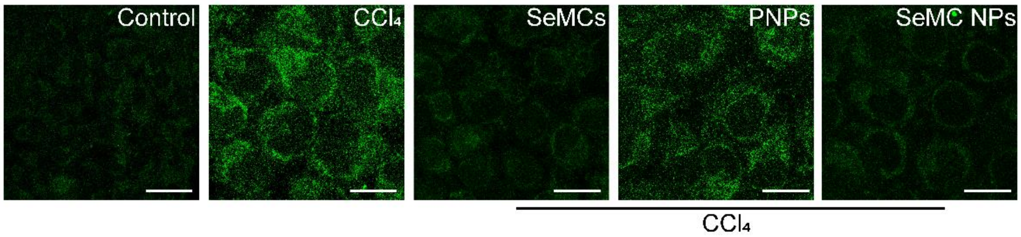

Figure S5 Effects of SeMC NPs on CCl_4-treated L-02 cells. Intracellular ROS levels in L-02 cells with indicated treatments detected by the DCFH-DA probe. Scale bar: 10 μm.

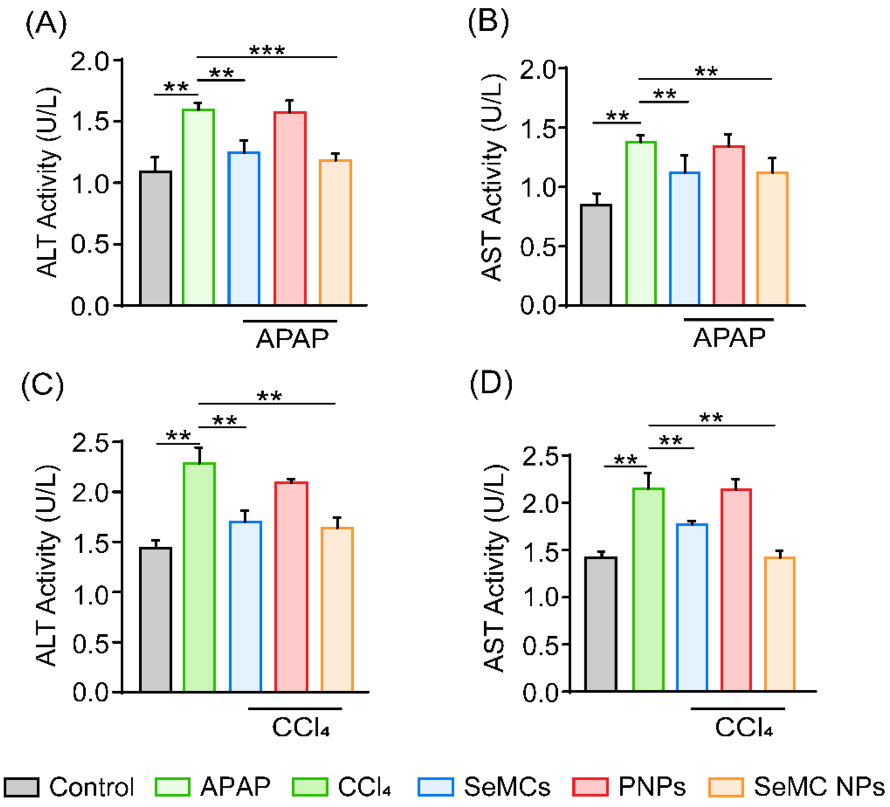

Figure S6 ALT and AST activities in L-02 cells. (A-D) ALT and AST activities in L-02 cells with indicated treatments. Data are represented as mean ± SD (n = 6). $^{**}p < 0.01$, and $^{***}p < 0.001$.

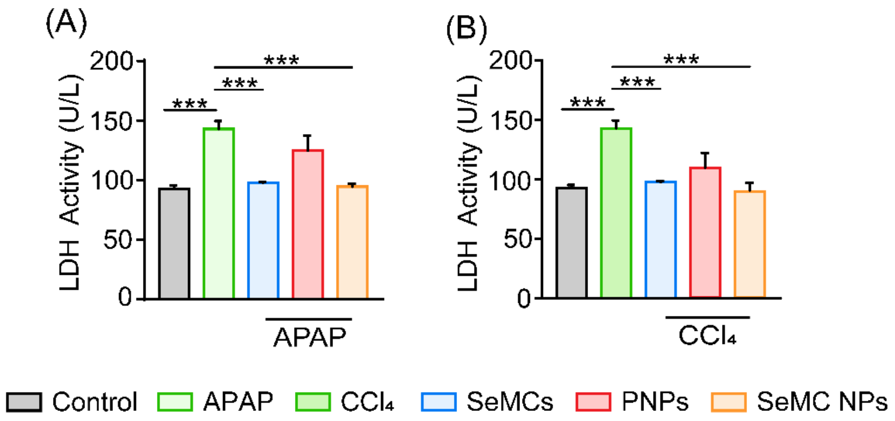

Figure S7 LDH activities in L-02 cells. Data are represented as mean ± SD (n = 6). **p < 0.01, and ***p < 0.001.

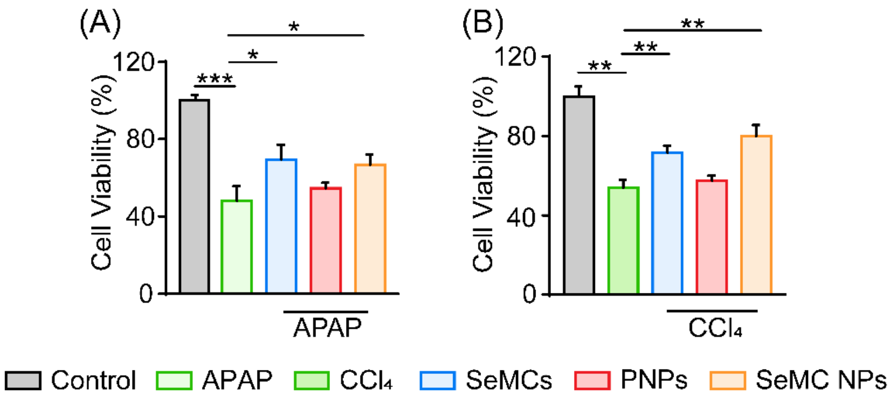

Figure S8 The protective effects of SeMC NPs on hepatocytes under various stimulations. (A-B) Cell viabilities of L-02 cells with indicated treatments. Data are represented as mean ± SD (n = 3). *$p < 0.05$, **$p < 0.01$, and ***$p < 0.001$.

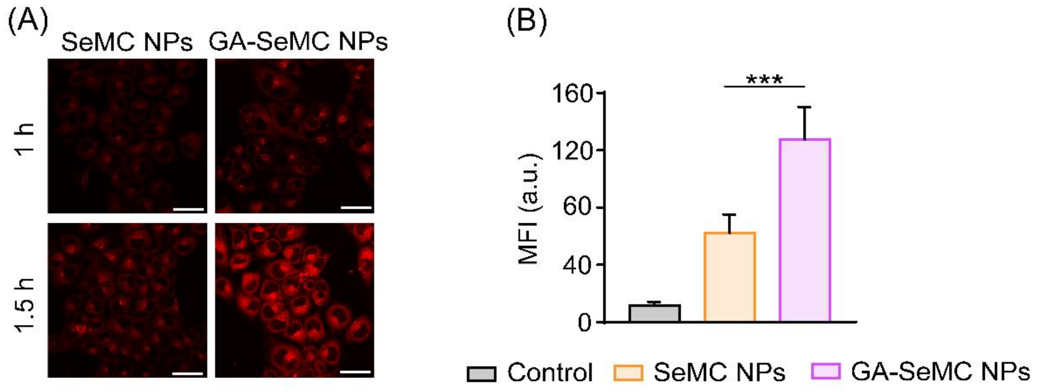

Figure S9 Targeting ability of GA-SeMC NPs *in vitro* and *in vivo*. (A) The representative fluorescence images of L-02 cells incubated with RhB-labeled SeMC NPs or RhB-labeled GA-SeMC NPs at the same RhB concentration at 37°C for 1 h and 1.5 h. Scale bars: 50 μm. (B) Quantitative analysis of the accumulation of RhB-labeled SeMC NPs or GA-SeMC NPs in liver. Data are represented as mean ± SD (n = 10). ***$p < 0.001$.

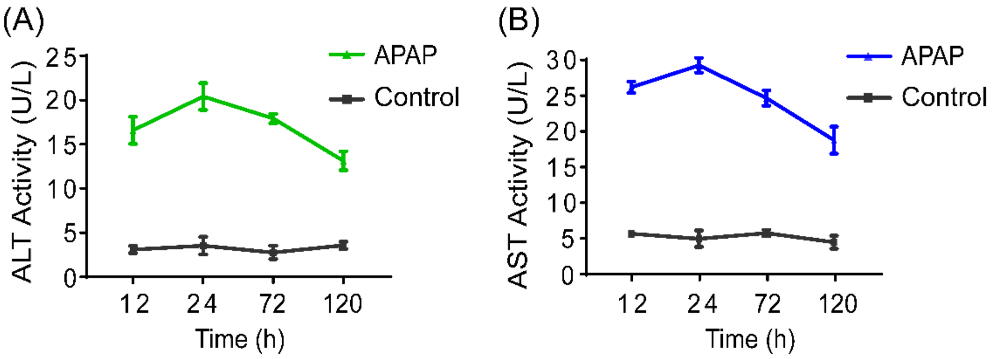

Figure S10 Establishment of a mouse model of APAP-induced ALI. (A and B) Serum ALT and AST levels in mice at the indicated time points after 300 mg kg^{-1} APAP injection. The control animals were injected with the same volume of PBS. Data are represented as mean ± SD (n = 6).

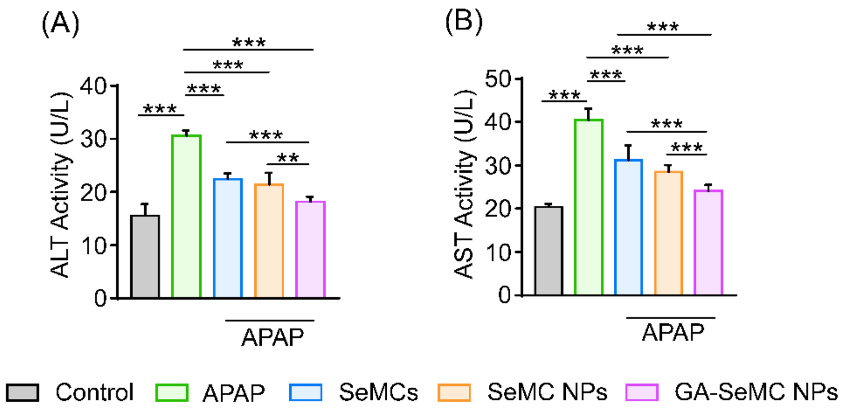

Figure S11 Effects of GA-SeMC NPs on the serum ALT and AST activities in APAP-induced ALI mice. (A and B) Serum ALT and AST indexes of mice in APAP-induced ALI mice with different treatments. Data are represented as mean ± SD (n = 6).

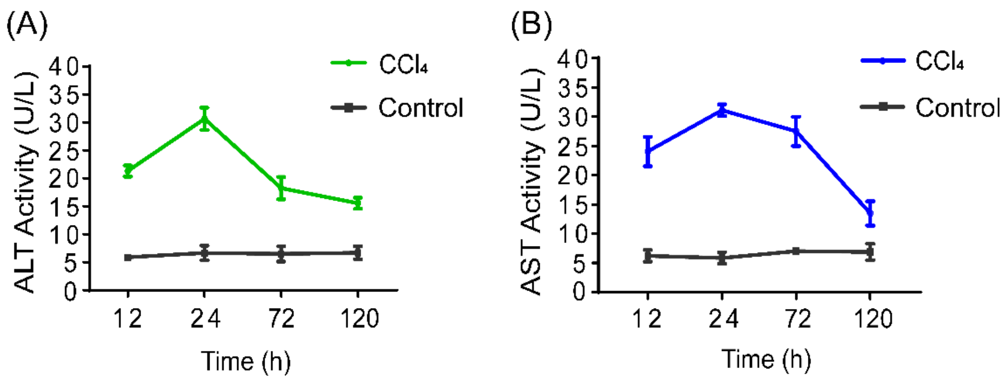

Figure S12 Establishment of a mouse model of CCl_4-induced ALI. (A and B) Serum ALT and AST levels in mice at the indicated time points after 10 mL kg^{-1} CCl_4 injection. The control animals were injected with the same volume of oil. Data are represented as mean ± SD (n = 6).

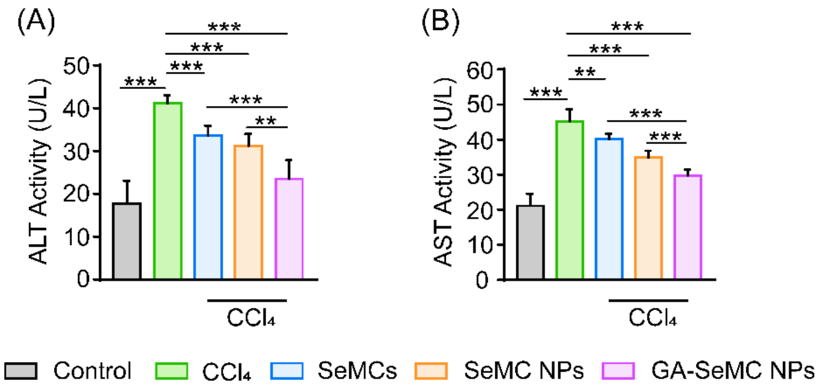

Figure S13 Effects of GA-SeMC NPs on the serum ALT and AST activities in CCl_4-induced ALI mice. (A and B) Serum ALT and AST indexes of mice in CCl_4-induced ALI mice with different treatments. Data are represented as mean ± SD (n = 6). $^{**}p < 0.01$, $^{***}p < 0.001$.
